# Youth peers put the “invent” into NutriBee’s online intervention

**DOI:** 10.1186/s12937-015-0031-2

**Published:** 2015-06-16

**Authors:** Ingrid C Kohlstadt, Elizabeth T Anderson Steeves, Kerry Rice, Joel Gittelsohn, Liane M Summerfield, Preety Gadhoke

**Affiliations:** 1 Center for Human Nutrition, Johns Hopkins Bloomberg School of Public Health, 198 Prince George St., Annapolis, Baltimore, 21401 MD USA; 2 University of Wisconsin, Milwaukee, WI USA; 3 Boise State University, Department of Educational Technology, Boise, ID USA; 4 Marymount University, Arlington, VA USA; 5 St. John’s University, College of Pharmacy and Health Sciences, New York, USA

**Keywords:** Peer group, Adolescents, Attitude to health, Food preferences, Nutrition, Service-learning, Chemosensory perception, Institute of Medicine, Diffusion of Innovations

## Abstract

**Background:**

Early adolescents perceive peers as credible and relatable. Peers therefore have a unique conduit to engage early adolescents in positive health behaviors through nutrition learning such as that recommended by the U.S. Institute of Medicine (IOM).

**Purpose:**

We developed an online, peer leader component to an existing in-person preventive nutrition intervention called NutriBee. We reasoned that youth ages 13–18 could create intervention materials that could remain engaging, credible and relatable to younger peers ages 10–12 online. Peer leaders could potentially derive health benefits from their service-learning experience.

**Methods:**

From 2013–2014 youth could apply online to relate a personal interest to nutrition, an opportunity promoted at NutriBee pilot sites and through social media. The peer leaders with diverse backgrounds honed original ideas into tangible projects with the support of adult subject-matter experts chosen by the youth. Nutrition expertise was provided by NutriBee staff who then also converted the youth-invented projects from various media into an online curriculum.

**Results:**

19 of 27 (70%) of selected youth from 12 states and diverse backgrounds, created an online curriculum comprising 10% of NutriBee’s 20-hour intervention. All 19 online projects modeled 1 or more of NutriBee’s 10 positive health behaviors; 8 evoked the chemosenses; 6 conveyed food texture; and 13 provided social context. Peer leaders perceived career advancement and service learning benefits. The dose, pedagogic approach, and project content align with the IOM recommendation.

**Conclusions:**

Youth created intervention materials which communicate positive health behaviors online in ways peers can adopt. In a customarily sight-sound digital platform, youth leveraged the senses of smell, taste and touch and social context important for food selection. Peer leaders derived health benefit, as indirectly assessed by IOM criteria.

## Introduction

In order to create impact, nutrition interventions for early adolescents must identify ways to make nutrition concepts relevant and engaging [1,2]. Incorporation of peers into intervention design and delivery is one strategy for achieving this, as peers are seen as a credible and reliable source of information [3], particularly slightly older (cross-age) peers [4]. Peers are especially important for early adolescents, as developmentally this is a timeframe in which youth are most susceptible to peer-influence [5].

Peers promote adoption of positive behaviors by helping younger peers connect learning of new material to prior experiences [6], make the abstract concrete [7] and, through collaborative learning, promote higher levels of problem solving [8]. Health interventions place increasing emphasis on narrative forms of communication such as storytelling, entertainment-education, and testimonials, approaches which lend themselves to leveraging peer-influence [9].

Peer-led nutrition interventions have demonstrated improvements in anthropometric measures [10-12], reduced consumption of snack foods and desserts [10], increased intake of fruits and vegetables [13] and improved psychosocial factors [11,12]. Peer leaders are often used to encourage uptake of novel foods by incorporating taste tests of food items as an intervention strategy [13-17].

Nutrition eHealth interventions are beginning to incorporate peer-leaders using social marketing [18] and video [19]. Electronic platforms can greatly expand reach [20,21], thereby enabling viewers with specialized interests to find compatible peers. However peer credibility of in-person interventions isn’t necessarily retained in the transition to electronic platforms. Nutrition interventions may be at a distinct disadvantage in the e-transition since food selection is heavily influenced by non-digital media such as olfaction, gustation, texture and social-cultural context [22], a challenge currently unaddressed in the education and medical research. Nutrition studies in a recent systematic review of electronic health interventions [20] were not peer-led. Furthermore, few peer-led nutrition interventions with strongly positive findings are electronic platforms, a finding which influenced the Institute of Medicine (IOM) to emphasize active learning approaches to nutrition [23].

Because of their inherent relatability and credibility, cross-age youth peers may be uniquely suited to serve as proxy for the taste, smell, texture of foods and the social milieu around which food selections are made. In theory youth-led intervention materials using electronic platforms could retain multisensory and social elements, which could be evaluated alongside behavior change constructs outlined by Diffusion of Innovations [24].

We developed a youth-led component to an existing preventive nutrition intervention called NutriBee [25], reasoning that youth ages 13–18 could creatively direct projects relating their personal interests to nutrition – projects that buttress NutriBee’s positive nutrition behaviors and retain elements online that make them credible and relatable to younger peers ages 10–12. A secondary research question is if the youth leaders could derive health benefit through their service-learning endeavors.

## Methods

**Figure 1:**
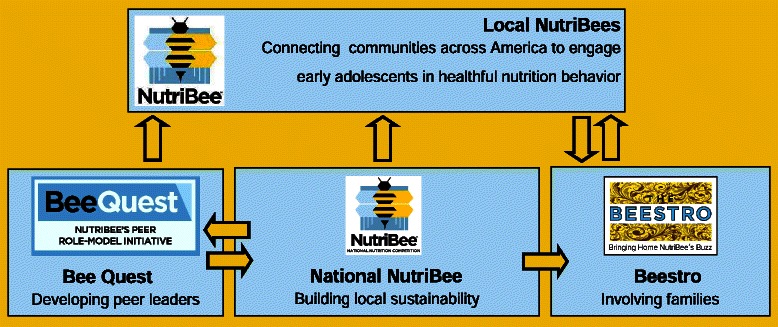
Bee Quest is NutriBee’s peer-led intervention where youth develop questions for the National NutriBee and online project materials for the otherwise in-person 20-hour nutrition intervention implemented in partnership with community programs.

NutriBee is a middle school-aged (10–12 years of age) nutrition intervention developed at The Johns Hopkins University Center for Human Nutrition to align with the nutrition education characterized by the IOM [23]. NutriBee’s primary intervention is a 20-hour IOM-aligned camp and club being disseminated nationally to early adolescents in partnership with community-based organizations and health care providers [25]. Among its engagement strategies are household reach thru child-as-change-agent activities aimed to strengthen impact at the household level impact, a planned national team-based bee-style game show, and the peer component called Bee Quest (Figure 1) [26].

Bee Quest involves high school-aged (ages 13–18 years) peers developing questions for the bee game through an original project (quest) that relates their interest, talent or hobby to nutrition. Promotional material and recruitment for Bee Quest emphasized that youth choose projects based on an activity they are passionate about, however remotely it may initially seem related to nutrition. While some examples were given, youth ultimately developed their own project ideas and were the creative directors, choosing the project deliverables and an adult coach as a subject-matter expert. Each project required three parts: Original project in the form of a video, slide presentation, essay, artwork, or computer application; a biosketch from the peer inventor(s); and a project-specific quiz called “Bee Questions” [26]. Developing quiz questions for the game show was intended to deepen the peer leaders’ nutrition knowledge and service-learning reflection.

Bee Quest 2013 recruitment was directed to NutriBee’s 9 pilot communities – ethnically-diverse, low-income areas in Maryland, Michigan, New Mexico and Guam – where community leaders held information sessions and sent emails. Bee Quest 2014 was promoted nationwide January through March of 2014 on NutriBee’s website, organizational list-serves (The Explorers Club, The Johns Hopkins Center for Talented Youth, Math Tree, Here There Everywhere Kids News), Linked-in nutrition interest groups, and word-of-mouth plus social media from past participants. The one-page applications were evaluated on the youth-coach team’s area-specific qualifications; project feasibility within the budget, timeframe and web-application parameters; and ability to expand nutrition’s perceived breadth. All completed applications were selected.

Upon project completion participants received a stipend, certificate, service learning credit and technical expertise for adapting their unique projects to NutriBee’s website. Coaches received a stipend, collaboration with NutriBee, access to resources and youth service-learning that directly benefits their organization.

A focus group taking place on 4/12/14 at the Cosmos Club in Washington DC was led by IK and involved 3 youth participants, 2 parents and 5 coaches (N = 10). The in-person focus group was limited by the program’s nationwide spread. Its aim was to generate discussion on the perceived benefits of participating in Bee Quest to guide the collection of process data.

Program metrics, media coverage, process evaluation, the focus group and educator assessment were used to assess Bee Quest on the following objectives:

### Diversity of topics and youth backgrounds

Geographic, gender, socioeconomic and ethnic diversity among peer leaders was reasoned to generate projects likely to be compatible with the correspondingly diverse early adolescent viewers.

### Service impact

The primary service is the youth projects presented online to be viewed by early adolescents. The 19 youth projects presented online were independently viewed by five (5) health and education professionals with the following backgrounds: Preventive medicine physician; doctor of social anthropology; nutrition education specialist who is also the vice president of academic affairs at a university with community service in its mission statement; university professor specializing in online and virtual education materials for early adolescents; and a doctoral candidate in behavioral health. The potential of the intervention materials to promote adoption of positive behaviors among early adolescent peers was characterized by the presence of the NutriBee’s predefined key health behaviors; inclusion of chemosensory, tactile and social elements; and the five (5) constructs comprising Diffusion of Innovations.

### Health impact on peer leaders

The health benefit to peer leaders was assessed indirectly by achievement of IOM recommendations for high school students [23]. These are an annual dose of 20 hours, hands-on (active) learning with adult guidance, and focused on nutrition and/or fitness.

### Service-learning benefit

Service learning benefit to peer leaders was assessed by process evaluation. Youth completing Bee Quest were asked how they felt that they had most benefitted, by selecting the 2 top benefits from the list of 5 developed with the focus group. Health benefits were not listed since these were indirectly assessed separately.

The Johns Hopkins Bloomberg School of Public Health IRB reviewed and approved NutriBee pilot research (IRB #4821) and the photo release form used for Bee Quest project participants.

Bee Quest webpage’s layout was designed in such a way that future NutriBee viewer audience’s interaction with the page can be analyzed using Google Analytics. Clickable thumbnail images of the projects are alphabetized by subject matter to minimize potential bias associated with page placement. A generic template (macro) was implemented to unify the projects’ various forms of media and styles, yet retain their originality.

## Results

Bee Quest participants hailed from 12 states and online projects were from NutriBee pilot areas. Participant photos accompany 18 (95%) of online projects and are anticipated to help connect with middle-school age viewers of diverse gender, race and ethnicity. The 27 project topics presented in Table 1 are varied: 5 (19%) art, 5 (19%) athletics, 4 (15%) culture, 4 (15%) ecology, 4 (15%) journalism, and 5 (19%) STEM (science technology engineering math).

**Table 1:** **Youth projects supported through Bee Quest 2013 and 2014**

2013	Project title	Category of student’s chosen topic
1	Food as symbolism in a Nigerian wedding	Culture
2	Sound nutrition: Theme music for NutriBee performed on kitchen instruments	Art
3	Nutrition-themed scavenger hunt at the Walters Art Museum in Baltimore	Art
4	I salute you, you feed me: Nutrition in the military	Journalism
5	Nutrition in action: Nutrition-themed field games	Athletics
6	Competitive gymnast interviews athletes on hydration	Athletics
7	What owl digestion teaches us about human nutrition	STEM
8	Not-so-trivial nutrition quiz kickball	Athletics
9	Fishing in the Anishinaabe tradition	Culture
10	Preparing an Ojibwe feast.	Culture
**2014**	**Project Title**	
1	Ballet: Nutrition expressed through movement	Athletics
2	Comic strip illustrating a recipe called Prime Number Parfait	Art
3	Card game for preteens promoting food selection and portion sizes	STEM
4	The origins of Peking Duck	Culture
5	An app for apt hydration	STEM
6	Transforming a dietary restriction into an opportunity	STEM
7	Good for you and good for your planet: A grocery store scavenger hunt	Ecology
8	An edible wild plant scavenger hunt in West Virginia’s mountains	Ecology
9	Geocaching for health	Ecology
10	Illustrating pet nutrition	Art
11	A journalistic news story on trending towards nutritious foods	Journalism
12	A news story on food blogs	Journalism
13	Pottery: Life extension for fruits and vegetables	Art
14	Nutrition-themed game for a baby shower	STEM
15	Wilderness food preparation for scouts	Ecology
16	Interviewing Verron Haynes, Superbowl XL Champion	Journalism
17	Running hydrated	Athletics

Of the 27 projects 25 (93%) contributed to NutriBee overall (Table 2) and 19 (70%) are featured on its webpage, where they collectively comprise 2 hours of online activity, 1/10^th^ of NutriBee’s intervention materials.

The educator and health professional reviewers (IK, PG, LS, EAS, KR) report their assessment in Table 3. Collectively the 19 projects address each of NutriBee’s 10 positive nutrition behaviors, and 2 or more of 5 reviewers found that all projects reference at least 1 of these 10 behaviors. Despite the conventional limitations of digital media, 2 or more of the 5 reviewers found that 8 (42%) projects modeled chemosensory aspects of food, 6 (32%) exhibited tactile interaction with food such as its texture or touch and 13 (68%) guide viewers through an aspect of the social milieu influencing food choices.

With Diffusions of Innovation constructs the five reviewers successfully characterized how each project is likely to lead to behavior change. In Table 3 projects were characterized in terms of: 1. Advantage of the new approach over the currently used one. 2. Simplicity. 3. Compatibility with viewers. 4. Observable results. 5. Triability which is how easily the approach can be experimented with before fully used.

**Table 2:** **Process evaluation parameters**

Parameter	n	Percent (%)
Project ideas submitted	27	100
Bee Quest 2013 and 2014 awarded projects	27	100
Projects completed	24	89
Projects incorporated into NutriBee online curriculum	19	70
Projects contributed to database of game show questions (Bee Questions)	25	93
Solo versus team projects	23	85
Project coach directs a youth program	16	59
Coach is a friend or relative of participant	9	33
Presented project in person at NutriBee pilot (2013 only)	5	50
Featured in the media	9	38

**Table 3:** **The health behaviors, sensory and social elements, and Diffusion-of-Innovation constructs which youth incorporated into their Bee Quest projects**

	Youth-developed Bee Quest Projects																		
	The abbreviations are the project topics as listed alphabetically on NutriBee.org/BeeQuest																		
	ac	ah	cg	ca	cc	cz	cp	ec	fb	ge	il	jo	mh	mu	np	po	ph	ru	sj
**NutriBee’s Key Health Behaviors**																			
N= 5: Experts independently reviewed the 19 projects for the presence of the following health behaviors.																			
Mindful eating	5	0	2	4	4	4	0	2	2	2	1	2	2	2	2	4	1	1	1
Balanced portions	0	0	5	2	0	0	0	2	0	0	0	5	0	0	1	1	4	1	2
Less sugar	1	2	3	0	0	0	0	2	1	1	0	5	0	0	1	1	2	4	5
More fiber	1	2	2	1	0	0	0	2	1	1	4	1	0	0	1	1	2	1	1
Plant-based	2	2	4	4	1	0	0	3	1	1	1	5	0	0	5	4	5	2	2
Better fats	1	0	1	1	0	0	0	2	1	3	1	4	0	0	1	1	1	0	5
Hydration	0	0	0	0	0	0	5	0	0	5	0	0	5	0	0	0	2	5	4
Breakfast	0	4	1	0	0	0	0	0	0	0	0	1	0	0	0	0	1	0	0
Connect food with nature	3	4	0	4	4	0	0	4	2	0	4	2	0	1	5	2	1	3	0
Food safety	3	0	4	0	2	0	0	4	2	4	0	0	0	2	1	5	5	1	1
**Multisensory Constructs**																			
N=5: Experts independently reviewed the projects for use of sensory and social elements.																			
Chemo-sensory	5	0	2	0	4	3	0	4	4	4	0	0	0	0	0	3	0	1	0
Touch and Texture	5	0	2	0	5	3	0	4	0	0	0	0	0	1	0	4	0	1	0
Social context	5	4	4	1	4	0	0	4	5	2	1	3	5	4	0	0	4	4	5
**Diffusion of Innovation Constructs**																			
The data is a sum ranging from 5-15: 5 reviewers independently considered the 19 projects using a 1-3 Likert scale, where 1 indicates that the construct was minimally represented and 3 indicated the construct was strongly represented.																			
Relative advantage	5	10	7	9	8	5	9	14	9	10	5	15	5	9	10	15	9	14	15
Simplicity	11	13	14	11	12	12	5	13	9	9	6	10	11	15	15	15	11	14	11
Compatibility	10	9	13	13	12	7	9	14	14	14	9	12	7	13	12	5	8	12	15
Observability	9	7	14	13	13	7	12	14	11	12	10	7	11	12	10	10	10	10	8
Triability	11	11	10	6	8	8	5	12	11	9	11	12	6	13	12	14	10	15	15

Secondary service impact was noted: Youth contributed 180 questions to NutriBee’s “Bee Question” database. Sixteen projects had coaches affiliated with youth organizations who received service benefit from the youth projects. Nine participants were interviewed or had their projects featured in the six newspaper and magazine articles citing Bee Quest [27-32].

Participants averaged 20 hours of project-related nutrition learning and received guidance from two or more adults, usually coaches, mentors, school teachers, parents or NutriBee’s team.

Focus group participants identified five benefits (Table 4), other than the health benefits assessed separately. All 22 (100%) Bee Quest participants responded to the process question on the two non-health benefits of participation that most related to them: Strengthening college application 17 (77%); service-learning 10 (45%); resources and coach 9 (41%); stipend 5 (23%); and original work featured on website 3 (14%). Having their original work featured on the website was of great interest to youth participants, but primarily as a way of strengthening their college application and helping people.

**Table 4:** **Bee Quest youth responses to benefits of participation**

Perceived benefits of participation	n	Percentage
Strengthen college application	17	77%
Help others while doing what I enjoy (service learning)	10	45%
Resources and coach to further develop my personal interest or career path	9	41%
Stipend (Amazon gift card)	5	23%
Original work featured on NutriBee’s website	3	14%

Participants selected the 2 of 5 benefits, other than health, which most applied to them.

All 22 (100%) participants responded.

The health impact of participating in Bee Quest was indirectly measured. Bee Quest meets the criteria for service learning which is a form of hands-on learning highly correlated with lifelong learning and behavior change [33]. The exposure level or dose was 20 hours of hands-on learning as recommended by the IOM [23], with at least two adults involved in guiding the youth participants’ learning process. The findings are corroborated by the five (5) parents and coaches attending the focus groups, and by the process evaluation respondents who indicated that nutrition’s relevance to their career choice and personal interests was strengthened.

Web analytics for NutriBee are as follows: from 9/1/14 thru 3/10/15 NutriBee had 3,037 views of which 2,143 (71%) were in the U.S. and 618 (11%) were visits to the Bee Quest webpage. Coding will need to be embedded into each student thumbnail project to assess project-specific analytics for future data collection.

## Discussion

The creative directorship from NutriBee’s peer leaders and the online accessibility of the youth-developed materials are benchmarks for peer-led nutrition interventions. Our findings support our primary research question, that ultimately the creativity and innovation modeled by the youth peer leaders would enable their service-learning projects to retain relatability and credibility when transferred to a digital platform. Peer-created intervention materials have potential to reinforce positive nutrition behaviors among early adolescents, based on their infusion of the health behaviors emphasized in NutriBee, retention of sensory and social elements, and the presence of Diffusion of Innovations adoption factors [24].

Other appropriate health behavior change models for nutrition include Social Cognitive Theory, Theory of Reasoned Action/Theory of Planned Behavior, Integrated Behavior Model, and Transtheoretical Model [34]. Bee Quest materials were not evaluated for social cognitive learning because they are intended as interventions rather than learning *per se*, and social learning theories stop short of explaining the motivations for learning which are ultimately what prompt behavior change [21]. Similarly, the Transtheoretical Model focuses upon stages of change for risky or addictive behaviors for adults and adolescents, rather than taking a broader, ecological approach to understanding factors influencing lifestyle nutrition, which are youth-relevant behaviors not pertaining to addiction medicine [35].

Involving youth peers in food-related chemosensory experiences has been successfully implemented in only a few in-person taste testing [13,15], no community gardening interventions [36,37], and one cooking intervention [19] published following a review article citing the lack of peer-led cooking interventions [38]. Further we found no eHealth or mHealth (mobile device) interventions specifically intending to evoke the senses that guide food selection beyond the otherwise sight-sound realm of electronic media [39-43].

The importance of chemosensory awareness during early adolescence was first underscored by a pediatrician, Marie Montessori [22]. A century later it is at the nexus of medical research ranging from basic neuroscience to social anthropology [44,45]. Clinical relevance is further punctuated by widespread use of prescription medications with nuanced, individualized and unknown yet preventable effects on food selection among youth [46,47]. Physicians are therefore uniquely positioned to encourage nourishing food selection among their early adolescent patients. Given the limited time that most physicians have with their patients, referral to easily-accessible online intervention materials is a preventive medicine tool meriting further study.

Our programmatic findings appear synergistic with the U.S. public health aim to implement 20 hours of hands-on learning in nutrition annually to school children including at the high school level (ages 13–18). The IOM recommendations represent a 10-fold increase in nutrition education, suggesting an urgent need for partnering organizations and leveraging resources. Since Bee Quest participants can be identified through health clinics as well as schools, health care professionals may want to recommend that their adolescent patients consider participating in future annual Bee Quests as a preventive medicine intervention.

In keeping with the Bee Quest students’ perceived benefit, service-learning is widely considered advantageous to otherwise equally qualified college applicants especially when service is part of the institution’s mission [48]. The American Association of Colleges and Universities (AAC&U) has identified service learning and community-based learning as one of 11 high-impact educational practices in higher education, because it allows students to apply classroom concepts to community problems and issues and to reflect on their service experience [48]. In Bee Quest high school students address the community issues surrounding junk food and chronic diseases for NutriBee and its virtual community of early adolescent peers. Bee Quest may be able to address the growing demand in U.S. high schools for service-learning opportunities [49], especially initiatives that attract high school talent into health professions, and can involve youth with learning or physical disabilities. Service learning opportunities in low income communities such as those reached by NutriBee are fewer but tend to have greater impact [50].

A study limitation is that the health impact on early adolescent viewers and youth participants has not yet been directly assessed. Researchers may implement and evaluate the program materials among early adolescents in their community or health center. Bee Quest intervention materials are open access [26] and a piloted NutriBee Youth Impact Questionnaire is available to assess health impact of engaging in the Bee Quest materials [25].

## Conclusions

Our youth-invented online projects model NutriBee’s 10 positive health behaviors, and contain elements which foster adoption of these behaviors by early adolescent viewers. Notably despite the conventional limitations of online materials, the intervention materials incorporate multisensory elements and social context increasingly important to clinical practice. Youth leaders derived health and career benefits through their service-learning projects, a synergy which could be further developed to include immigrants and youth with learning disabilities.
